# Time Trends in the Co-use of Cannabis and the Misuse of Tranquilizers, Sedatives and Sleeping Pills among Young Adults in Spain between 2009 and 2015

**DOI:** 10.3390/ijerph16183423

**Published:** 2019-09-15

**Authors:** Domingo Palacios-Ceña, Isabel Jiménez-Trujillo, Valentín Hernández-Barrera, Lidiane Lima Florencio, Pilar Carrasco-Garrido

**Affiliations:** 1 Department of Physical Therapy, Occupational Therapy, Rehabilitation and Physical Medicine, Research Group of Humanities and Qualitative Research in Health Science of Universidad Rey Juan Carlos (Hum&QRinHS), Universidad Rey Juan Carlos, Alcorcón, 28922 Madrid, Spain; domingo.palacios@urjc.es; 2 Preventive Medicine and Public Health Teaching and Research Unit, Health Sciences Faculty Universidad Rey Juan Carlos, Alcorcon, 28922 Madrid, Spain; isabel.jimenez@urjc.es (I.J.-T.); valentin.hernandez@urjc.es (V.H.-B.); 3 Department of Physical Therapy, Occupational Therapy, Rehabilitation and Physical Medicine, Universidad Rey Juan Carlos, Alcorcón, 28922 Madrid, Spain; lidiane.florencio@urjc.es

**Keywords:** marijuana abuse, cannabis, misuse, tranquilizing agents, sleep aids pharmaceutical, young adult, epidemiologic studies

## Abstract

The aims of this study were: (a) to estimate time trends in the prevalence of the co-use of cannabis and other cannabis-based products (CBP) with the misuse of tranquilizers, sedatives, and sleeping pills (TSSp) between 2009 and 2015; and (b) to identify the factors associated with the probability of the co-use of CBP with TSSp misuse during this period among Spanish younger adults (15–34 years old). We analyzed data collected from the Spanish National Surveys on Alcohol and Other Drugs (EDADES) in 2009, 2011, 2013, and 2015. CBP co-use with TSSp misuse were the dependent variables. We also analyzed sociodemographic features, self-perceived health status, lifestyle habits, perceived health risk of consumption, and perceived availability of substance using logistic regression models. The prevalence of CBP co-use with TSSp misuse has decreased in Spain. The factors associated with co-use were a lack of education (OR 2.34), alcohol (OR 7.2), tobacco (OR 6.3) and other illicit psychoactive drug (OR 6.5) consumption, perceived non-health risk for the consumption of CBP and TSSp (OR 3.27), and perceived availability of CBP (OR 2.96). Our study identified several factors that appear to affect CBP and TSSp co-use in younger adults, with potential implications for healthcare providers.

## 1. Introduction

Cannabis and other cannabis-based products (CBP) represent the most popular illicit drug in the Western world [1]. The cannabis plant produces a resin containing psychoactive compounds called cannabinoids [1]. The main psychoactive constituent of cannabis is delta-9-tetrahydrocannabinol (THC), the concentration of which varies across cannabis plants, depending on genetic factors, growing conditions, preparation, and extraction methods [2].

According to the United Nations Office on Drugs and Crime, in 2016, approximately 28 million adults in Europe aged 15 to 64 (5.1%) had used cannabis during the previous year [3]. However, the European prevalence of cannabis use varies significantly by country, with a higher prevalence in Mediterranean and Central-Western European countries, and a lower prevalence in Eastern and Northern European countries [4]. In the United States, during 2002–2014, the prevalence of marijuana use increased among persons aged ≥18 years [5]. Also, in 2014, a total of 2.5 million persons, aged 12 years or older, had used marijuana for the first time during the previous 12 months, with an average of approximately 7000 new users every day [5]. Moreover, in 2016, the illicit drugs with the largest number of recent initiates, aged 12 or older, were, in decreasing order: marijuana, prescription pain relievers, prescription tranquilizers, prescription stimulants, and hallucinogens, with rates ranging from 1.2 to 2.6 million new users overall [6].

The most frequent users are teenagers and adolescents [7] with problematic cannabis use typically peaking among the latter: a worrying trend as adolescents can be particularly vulnerable to the harmful side effects of cannabis [2]. This vulnerability to negative effects is also related to the presence of varying concentrations of the cannabis’s components. Cannabis markets are dominated by high-potency cannabis (high THC and low in cannabidiol concentrations), with the THC content steadily increasing worldwide [2]. Compared with low-potency cannabis, high-potency cannabis appears to be associated with a greater risk of psychotic symptoms, depression, anxiety, and cannabis dependency [2]. In addition, acute and chronic CBP use has been shown to be harmful with regard to several aspects of psychological and physical health, such as mood swings, psychiatric outcomes, neurocognition, driving, and general health. Furthermore, cannabis is highly addictive, and the adverse effects of withdrawal can lead to regular use [7,8]. As a result, the frequent use of CBP among young people has also been associated with disadvantages later in life, such as limited educational attainment, unemployment and lower life satisfaction [1,9]. Also, early initiation has been associated with a number of negative health and social outcomes, including a greater likelihood of dependence and problematic use, poor academic performance, the onset of substance use disorders, and adverse mental health outcomes [7,10,11].

Previous studies show how CBP use at a young age is associated with polydrug use combined with other substances, such as alcohol, tobacco, stimulants, and the misuse of prescription drugs, such as tranquilizers, sedatives, sleeping pills (TSSp), and depressants [11,12,13,14,15]. Research has highlighted the importance of age, attitudes, and beliefs as being important predictors of cannabis co-use with other substances among youth, such as perceptions of risk and the perception that CBP is easily available [9,16,17]. The co-use of other substances occurs following a “gateway sequence” phenomenon whereby the first-time use of a drug acts as a precedent or starter drug (gateway hypothesis) towards another illegal substance or the simultaneous use of various substances [11,12,18]. Other research studies have established that rational drug use occurs based on the safety of the substance providing the anticipated effects of psychological wellbeing (drug-substitution strategy) [19].

Substance co-use is associated with the risk behaviors and negative health outcomes outlined above, extending beyond the use of each individual substance in isolation. Also, young people who co-use substances tend to continue to do so as they age and are also more likely to take up additional substances rather than reduce the number of substances they use, over time [17]. Those who report wide-ranging CBP co-use are also more likely to report poor mental health, high-risk sexual behaviors, and substance use disorders in adulthood [17]. In Spain, the simultaneous use of multiple substances is common among adolescents and has increased during the last decade, including the combined use of sedatives and tranquilizers, along with alcohol, tobacco, and marijuana [13]. The prevalence and age of onset of consumption of cannabis were associated with the consumption of non-prescription sedatives, stimulants, and poly-consumption [14].

TSSp are medicinal drugs that are primarily prescribed for the treatment of anxiety, insomnia, sleep disorders, depression, and epilepsy. However, these drugs are also subject to misuse and abuse [15]. The use of TSSp with alcohol, amphetamines or cannabis is very common. [15]. Over recent decades, the prescription of psychotropic drugs has significantly increased among adolescents and young adults [20]. This increase in the number of prescriptions could result in increased misuse and associated consequences, owing to greater availability and the potential for abuse. Approximately 2.4% of American adolescents have misused prescription psychotherapeutic drugs (anxiolytics, sedatives, or hypnotics) [21]. Regarding the situation in Spain, alcohol and tobacco, followed by cannabis, remain the most highly consumed drugs used by Spanish adolescents. These are followed by hypnosedatives (with and without a prescription), cocaine, ecstasy, hallucinogens, amphetamines, volatile inhalants, and heroine, in this order [22]. Several studies, including research by Font-Mayolas et al. [23], suggest that Spanish adolescents, particularly males, start poly-drug use at an earlier age when compared to other European adolescents. Moreover, Carrasco-Garrido et al. [13] reported that the prevalence of TSSp misuse among students aged 14-18 years increased significantly from 2004 (2.40%) to 2014 (2.96%). As a result of cannabis co-use with the misuse of prescription drugs (TSSP) in young people, an increased risk of adverse effects, overdoses and traffic accidents has been identified [24,25,26].

Currently, there are no studies that describe the trend in the co-use of CBP and TSSp misuse among young adults in Spain. The objectives of the present study were as follows: a) to analyze time trends in the prevalence of CBP co-use with TSSp misuse among younger adults (15–34 years old) in Spain between 2009 and 2015; and b) to identify the sociodemographic features, self-rated health status, lifestyle habits, perceived health risk for consumption and perceived availability of substances associated with CBP co-use with TSSp misuse during this period among younger adults in Spain.

## 2. Materials and Methods 

### 2.1. Data Source

We conducted a nationwide, descriptive, cross-sectional epidemiological study on the co-use of CBP with TSSps misuse among individuals aged between 15–34 years in Spain. We used individualized secondary data drawn from the 2009, 2011, 2013, and 2015 Spanish National Surveys on Alcohol and Other Drugs, known as EDADES (Encuestas sobre Alcohol y Otras Drogas en España) [27,28,29,30]. 

Since 1995, the Spanish National Drug Plan has carried out a biannual national survey (EDADES) on individuals aged between 15 and 64 years old [29]. The EDADES is an ongoing survey that collects data by home-based personal interviews in order to examine a national representative sample of the non-institutionalized population residing in main family households in Spain. The surveys use multistage cluster sampling, with a proportional random selection of primary and secondary sampling units (towns and sections, respectively); the final units (individuals) are selected using random routes and gender- and age-based quotas. Surveyors were trained in basic communication skills, procedures, and administration of the questionnaire. Details of EDADES methodology are reported elsewhere [27,28,29,30]. 

The 2009 survey included 10,813 younger adults of both sexes interviewed between November 2009 and March 2010; the 2011 survey included 10,507 younger adults of both sexes interviewed between November 2011 and April 2012; the 2013 survey included 10,854 younger adults of both sexes interviewed between November 2013 and April 2014; lastly, the 2015 survey included 10,075 persons interviewed from December 2015 to April 2016. 

### 2.2. Variables

For the purposes of the present study, we included responses from young adults of both sexes aged between 15–34 years from the 2009, 2011, 2013 and 2015 surveys. All four surveys asked the same questions, allowing for comparison between the variables. 

The information used for creating the dependent variables (considered as dichotomous variables) was obtained from the “yes” or “no” answers to the following questions: “Have you taken marijuana, cannabis, or hashish during the last 12 months?” The questionnaire indicated that these cannabis products included: hash oil, “chocolate” and “costo” (Spanish popular terms for hashish), pot, grass. Also, “Have you taken a tranquilizer, sedative, and/or sleeping pill without a prescription during the last 12 months?” The questionnaire indicated that these drugs included: hypnotics, Trankimazin, Rohypnol, Tranxilium, Diazepam, Valium, barbiturates, Lexatin, Orfidal, Noctamid, benzodiazepines, zolpidem, etc. However, the following were not included: Valerian, Passiflora, and Dormidina (doxylamine). Also, we determined the co-use of CBP and TSSp misuse, when younger adults confirmed both questions. Co-use was considered to occur when, during the same period of time, the respondents used CBP and later TSSp, or vice versa.

The independent variables were the primary sociodemographic characteristics of the population—namely age, sex, nationality (Spanish or immigrant), occupational status (unemployed, employed, or inactive), and educational level (no education, primary school, secondary school, and higher education). To determine the use of other legal psychoactive substances, we used responses for alcohol consumption and smoking during the previous 12 months (dichotomous variable, yes/no). In order to determine the co-use of illegal psychoactive substances, there were questions on the consumption of drugs, other than marijuana (LSD, non-LSD hallucinogenics, amphetamines, cocaine, and heroin) during the previous 12 months.

We also used variables associated with the perceived risk of marijuana and other CBP, tranquilizers, sedatives, and consumption of sleeping pills. For the variable related to perceived risk, subjects were asked to provide their opinions on the health effects and other problems, that could result in CBP use, and tranquilizers, sedatives, and sleeping pill misuse. This variable was categorized as some/many problems or none/few problems (no or few problems/quite a few or many problems). The perceived availability to acquire the substances was categorized as impossible, very difficult/easy or very easy.

### 2.3. Statistical Analysis

We calculated the prevalence of total CBP consumption and TSSp misuse for each of the four surveys according to the study variables. All data analyses were performed separately for women and men. The Pearson’s χ^2^ test was used for the bivariate comparison of proportions, and statistical significance was set at *p* < 0.05 (2-tailed). 

To estimate the independent effect of each of the study variables on the consumption of CBP and TSSp misuse, we also obtained the corresponding adjusted odds ratio (AOR) by means of multivariate logistic regression analysis. All variables with a significant association in the bivariate analysis were included in the multivariate analysis, along with those variables that were considered relevant in the scientific literature. Once the model was constructed, we analyzed trends in CBP consumption and TSSp misuse during the study period and calculated the adjusted odds ratios (aOR). 

Estimates were made by incorporating the sampling weights and using the “svy” (survey command) functions of STATA (STATA Corp, College Station, Texas, TX, USA), which enabled us to incorporate the sampling design into all our statistical calculations (descriptive, χ^2^ test, logistic regression). Statistical significance was set at a 2-tailed α < 0.05.

The effective response rates in 2009, 2011, 2013, and 2015, were 50.1%, 50%, 50.3%, and 50.5%, respectively [27,28,29,30].

### 2.4. Ethical Statements

This article does not contain any study with human participants or animals performed by any of the authors. In accordance with Spanish legislation, and accordingly, there is no need for Ethics Committee approval, since the database was obtained from the webpage of the Spanish National Drug Plan, EDADES surveys [31], where it is publicly available. All the surveys analyzed were anonymous, dissociated and contained no recognizable personal information. All content is in accordance with the second paragraph, section 5 (Orden SAS/3470/2009, 16 December) and does not fall within the assumptions established in Article 2.e (Law 14/2007, 3 June) concerning biomedical research.

## 3. Results

A total of 42,249 young adults from the EDADES survey during 2009–2015 were included, of these, 225 (0.56%) presented co-use of CBP and TSSp misuse. The study population comprised 140 men (0.71%) and 85 women (0.4%), of whom 26 (0.42%) were aged 15–18 years, and 199 (0.58%) were aged 19–34 years. The first time the respondents used CBP was at an average age of 17.19, while the onset of TSSp misuse was reported at an average age of 21.95. Moreover, the total prevalence of consumption CBP decreased from 2009 (*n* = 2206; 19.31%) to 2015 (*n* = 1719; 16.65%). The same decrease was observed regarding the consumption of TSSp in the same years (n = 181; 1.84% versus *n* = 141; 1.42%) respectively.

The co-use of CBP and TSSp misuse decreased during 2009–2015 in both sexes, reaching 0.37% in men (*p* = 0.000) and 0.3% in women (*p* = 0.360). See Table 1. 

Co-use decreased significantly in young adults aged 19–34 years between 2009 and 2015 (*p* = 0.000). There were differences in the age of onset of CBP consumption and TSSp misuse. Cannabis-based products reported a significant decrease in young adults <15 years (*p* = 0.007) and those aged between 16–18 years (*p* = 0.002). Additionally, TSSp misuse showed a decrease over 18 years (*p* = 0.001). Consumption levels also decreased significantly (*p* < 0.05) during 2009–2015 in both Spanish and immigrant individuals, in the case of respondents who were both employed and unemployed, and among individuals with secondary school and higher education. In addition, consumption decreased between 2009 and 2015 among young people who consumed alcohol, tobacco, CBP, TSSp (*p* = 0.000), and other illicit psychoactive drugs (*p* = 0.017) during the previous 12 months. Moreover, the number of young adults who had no or few problems perceiving the health risk associated to consumption of CBP, and who perceived the availability of CBP as being easy or very easy during the previous 24 h decreased significantly between 2009–2015 (*p* = 0.000).

Furthermore, there were differences in the age of onset of CBP and TSSp misuse. A significant decrease of CBP was reported among young adults <15 years (*p* = 0.007) and youth aged between 16–18 years (*p* = 0.002). Likewise, TSSp misuse decreased in young adults over 18 years of age (*p* = 0.001).

The results of the multivariate analysis are shown in Table 2. In our sample, male gender tended to be a predictor of the simultaneous consumption of CBP and TSSp (AOR, 1.22; 95% CI, 0.87–1.70) and being aged between 19–34 also tended to be a protective factor compared to individuals aged 15–18 years old (AOR 0.97; 95% CI: 0.59–1.61). When the consumption pattern among young adults was analyzed, the variables that were independently and significantly associated with a probability of simultaneous consumption of CBP and TSSp misuse were the lack of education (OR, 2.34; 95% CI, 1.01–5.44), alcohol use (OR, 7.2; 95% CI, 2.42–11.41), smoking cigarettes (OR, 6.3; 95% CI, 3.92–10.02), and the use of illicit psychoactive drugs other than CBP in the previous 12 months (OR, 6.5; 95% CI 4.63–9.02).

Furthermore, health profile analysis showed that the following factors were all significantly associated with a more frequent simultaneous consumption of CBP and TSSp: reduced or no perception of the health risk of consuming CBP and TSSp, perceiving the availability of CBP as being easy or very easy in the last 24 hours, and reported self-assessment of health status as being fair/poor/very poor.

The analysis of trends in Co-use of CBP and TSSp misuse during 2009–2015 revealed that the aOR was 0.87 (95% CI 0.81–0.94). The co-use of CBP and TSSp misuse among Spanish young adults decreased during the study period.

## 4. Discussion

This is the first study to report the prevalence and risk factors associated with co-use of CBP and TSSp misuse among Spanish young adults. Our results showed that the prevalence of CBP co-use with TSSp decreased in Spain between 2009–2015. The factors associated with co-use were no formal education; alcohol, tobacco and other illicit psychoactive drug consumption; not perceiving consumption of CBP and TSSp as being a health risk, easy availability of CBP in the last 24 hours, and poor self-assessment of health status.

The authors were unable to find any previous studies describing and analyzing the trends of co-use CBP with TSSp misuse. Thus, no comparison could be made regarding the growth or decrease of co-use of both substances in other studies and countries. Separately, CBP and TSSp consumption presented different trends. Prescription of TSSp has increased significantly among adolescents and young adults [13,20]. Furthermore, there seems to be a consensus about the decrease of CBP consumption among adolescents over the decade between the early 2000s and 2015 in Europe, Canada, and the United States [9,32,33]. However, this tendency towards a decrease could be modified by the legalization of CBP. After the Canadian government legalized recreational cannabis use in October 2018, Zuckermann et al. [34], reported that, after a steady decrease in patterns of cannabis use among young people over several years, it appears that there has been a gradual increase in cannabis use among youth, with some segments of this population being at greater risk. In another study, the same research team [17] suggested that the recent legalization of cannabis in Canada may increase the risk of poly-substance use (alcohol, cigarettes, cannabis, and e-cigarettes) confirming a significant increase of this phenomenon over time. The current debate surrounding the legalization of marijuana and its consequences (legal, health and social) highlights a controversial issue which should not be minimized [35]. In the United States, although the federal law forbids the use of CBP, its use is commonplace [36]. In addition to the federal law, state medical marijuana laws (currently 28 states have followed these regulations after the California bill was passed) and state recreational marijuana laws (Colorado, and six other states) enable the use of CBP [37]. This translates to the appearance of a legalization policy (meaning that a legal supply of CBP is allowed, together with the removal of penalties for the possession of small amounts of cannabis for recreational use) or decriminalization policy (thus, the penalty for the possession of cannabis is comparable to small traffic violation fines) [37]. Currently, there is a lack of wide and generalized information providing data on the relationship between the approval of laws which enable CBP consumption with changes on the prevalence of consumption and its consequences [37]. Previous studies [36,37,38] have reported how legalization for recreational use can pose many disadvantages, such as increased consumption and health related problems, when decreasing the price of purchase—as a result, this can encourage lucrative interests among companies (that commercialize CBP) above public health priorities, and can offer opportunities for the legal use of the same substance in a wider population by removing the need for a health professional (doctor) to prescribe and approve its consumption under certain circumstances. Nonetheless, several advantages may be noted, such as the benefits to social justice by removing certain mechanisms for race inequalities (drug trafficking) and the inconsistent application of the law on minorities in situations of vulnerability (poverty) [37,38]. Other benefits include savings on public money spent on the application of the law and the judicial system and increasing the income to state and local governments, via taxes [37,38]. 

In our sample, we identified that being aged between 19–34 years old is a protective factor of co-use in comparison to young adults aged between 15–18 years old. However, this finding cannot be generalized considering the confidence interval of OR (0.59–1.61). The findings of our sample may be related to the probability of young adults (>18 years old) being employed, thus decreasing their economic vulnerability, which could also be a protective factor against substance use [39,40]. In contrast, Arias-De la Torre et al. [14] described how Spanish college students (>18 years) showed a high consumption of cannabis with no prescribed sedatives, and a poly-use of cannabis with other substances, such as alcohol.

A gender related risk was also identified in our sample, with a greater risk for CBP and TSSp co-use observed in males (OR = 1.22). However, the limits of the confidence interval do not allow consistent external extrapolation (OR CI 95% = 0.87–1.70). Furthermore, the association between male gender and drug use has already been demonstrated (REFS). Previous studies [14,17,32], reported a significant association between men and a higher prevalence of CBP consumption, and a greater increase in CBP use along with a greater increase of cannabis related disorders. Male students were also significantly more likely to report poly-substance use [17]. Moreover, men were significantly more likely than women to try drugs and to be polyconsumers, with the exception of non-prescription sedatives [14], although, in the same study, the use of cannabis and non-prescription sedatives was notable in both genders, and everyone reporting poly drug use had tried cannabis [14]. 

Our results did not reveal differences in patterns of CBP and TSSp co-use between immigrant and autochthonous young adults. Previous studies conducted in Spain [41,42] did not identify differences in the use of illicit drugs or prescription drugs between immigrant and autochthonous young adults. Sarasa-Renedo et al. [42] reported how among immigrant adolescents the main factors mediating adolescents´ lower risk were less frequent socialization in leisure environments and less association with peers who use such substances. In this case, cannabis, tobacco, and stimulant use in immigrant adolescents increased with the increasing population use of these substances in the country-of-origin. 

The increased likelihood of using CBP and TSSp associated with alcohol, tobacco, and other drugs supports previous reports [13]. McCabe et al. [20] reported that the probability of using marijuana was almost 14-fold greater in respondents who reported misuse of benzodiazepines and anxiolytics (OR 13.7; 95% CI, 10.4–18.0). Carrasco-Garrido et al. [13] reported that the variables associated with a greater probability of TSSp misuse were the consumption of alcohol, tobacco, and marijuana (OR 1.52, 1.33–1.74 CI 95%; OR 1.33, 1.17–1.51 CI 95%; OR 1.41; 1.23–1.61 CI 95%, respectively). In addition, Fedorova et al. [43] showed that illicit drug use was associated with use of cannabis concentrates (OR 2.8, 95% CI 1.6–4.9), and prescription drug misuse increased for participants who reported use of cannabis edibles (OR  2.0, 95% CI 1.1–3.5). 

Potential explanations for the poly drug use, are offered by the gateway hypothesis [12] and the drug-substitution strategy [19]. The first hypothesis states that substance use follows a sequence of predicted drug use, beginning with alcohol and tobacco use, followed by cannabis use and, ultimately leading to other illicit drugs [12]. Thus, according to this hypothesis, a person rarely uses certain substances, such as heroin, without having first used certain “gateway” substances, such as alcohol or cannabis. Hence, cannabis fulfills the conditions for being a gateway drug if (a) its use begins before initiating use of other illicit drugs, and (b) if cannabis consumption increases the probability of using other illicit drugs [12]. Secades-Villa et al. [12] reported that 44.7% of individuals with lifetime cannabis use progressed to other illicit drug use at some time in their lives. Thus, during the second year after initial cannabis use, the probability of beginning the use of other illicit drugs was 8.7%. In addition, the estimated cumulated probability of other illicit drug initiation ten years after the onset of cannabis use was 36%. The drug-substitution strategy is based on an assumption that most people use lifestyle drugs rationally for self-medication purposes, to achieve specific desired psychological effects (well-being) [19]. When there is access to an equally effective, but (perceived) safer, alternative drug, then people tend to switch to it (especially when the substitute is legal and socially acceptable). There are several perceived safer lifestyle drug-substitutes for alcohol, including benzodiazepines, SSRIs, and marijuana [19]. An added risk is associated with poly drug use, because the cannabis consumption patterns have changed [44]. Coincident with cannabis legalization in some regions and the increased interest in medicinal use of the plant, there is now an expansive retail cannabis marketplace with emergent cannabis product chemotypes (e.g., THC-dominant, CBD-dominant, balanced or ‘hybrid’ with high concentrations of THC and CBD), product formulations (e.g., edibles, concentrates), and methods of administration (e.g., smoked, vaporized, orally ingested) [44]. Peters et al. [45] reported how CBP legalization and commercialization have introduced novel alternative cannabis products (edible and vaporized cannabis) and its association with poly drug use (use of ≥2 different products).

Indeed, our results reveal that having a lower risk perception, and perception of easy availability of substance were more predictive of co-use of CBP with TSSp misuse. These results are consistent with previous studies, in that a significant association was identified with a low perception of risk of cannabis and TSSp consumption and the perception of easy availability [5,13,16]. The perceived risk and availability related to several substances have evolved over the last decade. The prevalence of adolescents who perceive greater risk from smoking marijuana once or twice a week has decreased [5,16] and the prevalence of no perceived risk has increased [5,16]. An increase has also been demonstrated for the percentage of individuals reporting that marijuana was fairly easy or very easy to obtain [5]. In the case of TSSp, Carrasco-Garrido et al. [13] showed that the low perceived risk among high-school students in Spain, regarding the misuse of TSSp, makes them three times more likely to consume these drugs than adolescents who perceive consumption as high-risk. This observation is in line with the results of a study based on a national survey of Brazilian adolescents who reported that, compared with adolescents who perceived a high risk for the misuse of tranquilizers and sedatives, those who perceived a low risk (OR = 1.53; 95% CI,1.09–2.15) were also more likely to consume these drugs [46]. This finding, together with the ease with which TSSp can be obtained by adolescents (5.2% report that the drugs are easy or very easy to obtain), worsens the problem, as a significant association with consumption of TSSp.

Despite the decrease in the use of CBP and/or TSSp observed in the current study, or even, in order to maintain this trend, public strategies are required in order to disseminate the risk, clarify those potential benefits, and to monitor the availability related to these substances. One major goal of substance use prevention programs is to prevent or delay the initiation of substance use (i.e., first use) [6]. A young adult’s perception of the risks associated with substance use is an important determinant of whether he, or she, engages in substance use [16]. On the other hand, in many communities, cannabis is perceived as a low-risk drug, leading to political (and publicity messages in mass media and social networks) lobbying to decriminalize its use [8]. Although cannabidiol (CBD), one of the most important cannabinoid components, has been shown to have positive health outcomes, high-potency cannabis is particularly damaging, due to its high THC, and low CBD concentration. It is this high-potency substance that is readily available recreationally. At present, young adults have access to cannabis concentrates with a higher percentage of THC (69%), very different from the THC concentration of other products such as herbal cannabis (1.8%–5.7%), or resin (0%–29.3%) [2]. While research initiatives continue to investigate the medical benefits of CBD, “medicinal cannabis” still contains damaging levels of THC [8,33]. As a result, the increase in consumption based on inadequate or scarce information could facilitate the presence of new risks in the young population [33].

Despite divergences in the increased prevalence of the consumption of CBP in the USA and in Europe [32,33], the authors believe that there is a need for further prevention programs for CBP, as it continues to be a current and continuous problem. The programs must be directed at explaining and demonstrating the health risks of the consumption of CBP, together with the poly-use of other substances, with the aim of offering a true perspective of the risks of its consumption [47]. For this purpose, it is necessary for health bodies to use the same means of communication that adolescents and youth use, such as internet social networks, for health education campaigns. Health education must cover aspects such as the different substances that comprise CBP, the diversity in the concentration of TCH and CDB, the different forms of consumption and the explanation on the “medicinal use” of CBP. Furthermore, monitoring programs are required to research the consumption of CBP in homes and secondary schools (surveys), changes in the sale of cannabis, the amount of legally produced cannabis plants; and the THC content of cannabis, indicators of damage related with cannabis, such as deaths and injuries because of automobile accidents, emergency admissions, care in services for treatment of addictions and mental health, and the prevalence of the regular consumption of CBP among adolescents and youth [36,48].

It is necessary to continue monitoring the young population between 15–18 years, and the co-use of CBP and TSSp, along with alcohol and tobacco consumption. However, this poses a challenge because of monitoring and intervention difficulties. Cannabis-based products and TSSp present different classifications based on their medical value, the risk of addiction, and ability to cause harm [49]. In the United States, the Controlled Substance Act established five drug schedules. The schedules range from schedule I (most potential for addiction and use disorder) to schedule V (least potential for addiction/use disorder). Thus, cannabis (legalized or not) is classified as Schedule I, while some sedatives and tranquilizers are included in Schedule II, and some hypnotics such as benzodiazepines in Schedule IV. Thus, access, risk perception, and potential misuse (in legalized substances that require prescription), are different and require different approaches, by multiple professionals and different areas of intervention (health, social, legal and security).

### Limitations

This study has several limitations. First, we used a self-reported measure of CBP and TSSp co-use, which may have limited the assessment of misuse or abuse. Nevertheless, even though individuals can overestimate or underestimate their substance consumption, surveys are extremely useful for investigating patterns, frequencies, and longitudinal trends of CBP consumption and TSSp misuse. The sociocultural characteristics surrounding drug use may have led some young adults to be reluctant to admitting to the consumption of CBP and/or TSSp with or without a prescription. Also, some replies may be socially conditioned owing to the sociocultural association between psychotropic drug use and mental health problems, or CBP use and illicit drugs and illegality in general. Second, we did not consider populations over 34 years old. Third, the study design did not enable us to establish a cause and effect relationship owing to the lack of a longitudinal follow-up. Nevertheless, the use of a national population-based survey makes it possible to include representative national sample sizes. Despite these limitations, our study provides additional insight into demographic aspects, perceived health risk concerning consumption, and perceived availability of CBP substance co-use with TSSp misuse in younger adults, for whom there is little information at the population level, particularly in Spain.

## 5. Conclusions

The current study revealed a decrease in the co-use of CBP and TSSp misuse in Spain between 2009 and 2015. The identified factors associated with greater consumption were lack of education, use of alcohol, tobacco and other illicit psychoactive drugs, a lack of perceived health risk concerning consumption of CBP and TSSp, perceived availability of CBP (easy/very easy to obtain), and fair/poor/very poor self-assessment of health status. Our results have clear implications for health services in Spain. Health services should develop joint programs focused on the prevention of CBP use, appropriate TSSp prescription and TSSp misuse, and monitor the consumption of CBP and TSSp in younger adults <18 years.

## Figures and Tables

**Table 1:** Prevalence of co-use of cannabis and other cannabis-based products (CBP) and tranquilizers, sedatives, and sleeping pills (TSSp) misuse in young adults of both sexes, aged 15 to 34 years in Spain, according to sociodemographic variables, use of licit and illicit psychoactive drugs and variables related with perceived health risk, perceived availability and CBP and TSSp consumption ages. EDADES survey 2009–2015.

		2009	2011	2013	2015	Total	*p*-Value
		*n* (%)	*n* (%)	*n* (%)	*n* (%)	*n* (%)	
Sex	Male	61 (1.29)	35 (0.69)	25 (0.45)	19 (0.37)	140 (0.71)	0.000
	Female	32 (0.49)	15 (0.33)	23 (0.48)	15 (0.3)	85 (0.4)	0.360
Age	15–18	13 (0.67)	5 (0.43)	6 (0.31)	2 (0.19)	26 (0.42)	0.062
	19–34	80 (0.95)	45 (0.53)	42 (0.49)	32 (0.36)	199 (0.58)	0.000
Nationality	Spanish	78 (0.87)	48 (0.61)	44 (0.53)	31 (0.35)	201 (0.59)	0.000
	Immigrants	15 (1.02)	2 (0.04)	4 (0.14)	3 (0.26)	24 (0.39)	0.027
Occupational Status	Employed	34 (0.89)	14 (0.33)	18 (0.42)	12 (0.26)	78 (0.49)	0.003
	Unemployed	29 (1.39)	25 (1.14)	13 (0.49)	14 (0.68)	81 (0.91)	0.008
	Inactive	30 (0.72)	11 (0.32)	17 (0.54)	8 (0.27)	66 (0.45)	0.071
Educational Level	No studies	5 (2.37)	2 (0.6)	0 (0)	2 (1.48)	9 (1.38)	0.481
	Primary School	17 (1.04)	7 (0.47)	7 (0.88)	5 (0.44)	36 (0.74)	0.268
	Secondary School	58 (0.84)	29 (0.46)	33 (0.43)	23 (0.35)	143 (0.52)	0.002
	Higher Education	10 (0.66)	12 (0.79)	8 (0.42)	4 (0.15)	34 (0.51)	0.011
Alcohol Use in the Past 12 Months	Yes	88 (1.09)	50 (0.66)	48 (0.58)	34 (0.43)	220 (0.69)	0.000
Any Cigarette Smoking in the Past 12 Months	Yes	92 (1.28)	50 (0.77)	46 (0.67)	32 (0.51)	220 (0.82)	0.000
Any CBP Use in the Past 12 Months	Yes	93 (4.66)	50 (3.04)	48 (2.81)	34 (2)	225 (3.2)	0.000
Any TSSp Misuse in the Past 12 Months	Yes	93 (48.97)	50 (42.76)	48 (42.77)	34 (23.51)	225 (40.17)	0.000
Any Illicit Psychoactive Drug Use, Other than CBP, in the Last 12 Months	Yes	62 (6.19)	30 (5.81)	15 (3.28)	19 (4.21)	126 (4.96)	0.017
Perceived Health Risk for Consumption of CBP	No/Few Problems	62 (2.29)	36 (1.63)	25 (1.08)	23 (0.91)	146 (1.5)	0.000
	Quite Few/Many Problems	31 (0.38)	14 (0.17)	23 (0.27)	11 (0.15)	79 (0.24)	0.004
Perceived Health Risk Associated to Consumption of TSSp	No/Few Problems	32 (1.28)	26 (1.04)	16 (0.67)	12 (0.51)	86 (0.88)	0.063
	Quite Few/Many Problems	61 (0.73)	24 (0.3)	32 (0.38)	22 (0.28)	139 (0.43)	0.000
Perceived Availability of CBP in the Last 24 Hours	Impossible/Very Difficult to Obtain	5 (0.31)	2 (0.04)	1 (0.04)	4 (0.17)	12 (0.13)	0.470
	Easy/Very Easy to Obtain	87 (1.17)	48 (0.73)	47 (0.65)	30 (0.43)	212 (0.75)	0.000
Self-Assessment of Health Status	Fair/Poor/Very Poor	27 (3.22)	10 (2.04)	11 (1.65)	9 (1.16)	57 (2.1)	0.015
	Very Good/Good	66 (0.72)	40 (0.42)	37 (0.38)	25 (0.29)	168 (0.45)	0.001
**Total**		93 (0.9)	50 (0.52)	48 (0.47)	34 (0.33)	225 (0.56)	0.000

CBP = cannabis-based products; TSSp = Tranquilizers, sedatives, and sleeping pills.

**Table 2:** Multivariable logistic regression of factors associated with co-use of cannabis and other cannabis-based products and misuse of tranquilizers, sedatives, and sleeping pills in young adults of both sexes, aged 15 to 34 years in Spain. EDADES survey 2009–2015.

		OR	CI 95%
Sex	Female	1	
	Male	1.22	(0.87–1.70)
Age	15–18	1	
	19–34	0.97	(0.59–1.61)
Educational level	Secondary School	1	
	No Studies	2.34	(1.01–5.44)
	Primary School	1.44	(0.91–2.30)
	Higher Education	1.09	(0.70–1.70)
Alcohol Use in the Past 12 Months	Yes	7.2	(2.42–11.41)
Any Cigarette Smoking in the Past 12 Months	Yes	6.3	(3.92–10.02)
Any Illicit Psychoactive Drug Use Other Than CBP in the Last 12 Months	Yes	6.5	(4.63–9.02)
Perceived Health Risk Associated to Consumption of CBP and TSSp	Quite few/many problems	1	
	No/few problems	3.27	(2.31–4.62)
Perceived Availability of C&PCP	Impossible/very difficult to obtain	1	
	Easy/very easy to obtain	2.96	(1.49–5.89)
Self-Assessment of Health Status	Very good/good	1	
	Fair/poor/Very poor	3.51	(2.42–5.08)
Surveys		0.87	(0.81–0.94)

CI = Confidence Interval; CBP = cannabis-based products; OR = Odds Ratio; TSSp = Tranquilizers, sedatives, and sleeping pills.
